# The Gut-Brain Axis: A Case Report of Transanal Extrusion of a Ventriculoperitoneal Shunt and Literature Review

**DOI:** 10.7759/cureus.106508

**Published:** 2026-04-06

**Authors:** Marissa C Kuo, Andrew C Kuo, Jeffrey R Thomas, Jason A Jaffe, Christian Schuetz, Paul C Kuo

**Affiliations:** 1 Surgery, Vanderbilt University Medical Center, Nashville, USA; 2 Surgery, Sarasota Memorial Hospital, Sarasota, USA; 3 Surgery, University of South Florida Morsani College of Medicine, Tampa, USA

**Keywords:** anal extrusion, bowel erosion, laparotomy, primary repair of colon, ventriculoperitoneal shunt

## Abstract

Ventriculoperitoneal shunts (VPS) are widely used for the management of hydrocephalus but may rarely cause spontaneous bowel perforation, a potentially life-threatening complication associated with significant morbidity and mortality. This report describes an 81-year-old male who presented with transanal extrusion of VPS tubing six months after shunt placement, without abdominal pain, peritonitis, or systemic signs of infection. Imaging demonstrated catheter perforation of the descending colon with intraluminal migration to the rectum. The patient underwent multidisciplinary management, including shunt removal, exploratory laparotomy, primary colonic repair, and targeted antimicrobial therapy, with an uncomplicated recovery. To contextualize this case, a focused literature review identified 30 reported adult cases of VPS-associated spontaneous bowel perforation. Review findings indicate that cases in adults often progress slowly, with a substantial proportion identified by chance or after catheter protrusion without any abdominal symptoms, and a relatively low overall mortality rate, largely due to infections. Proposed risk factors include bowel wall compromise from chronic inflammation, prior surgery, or diverticular disease, while chronic catheter irritation may contribute to gradual erosion and contained perforation. Management strategies reported in the limited literature available vary significantly and include shunt externalization, endoscopic or laparoscopic catheter removal, and laparotomy in cases of peritonitis or instability. This case highlights the importance of maintaining clinical vigilance for VPS-related bowel perforation in adults, even in asymptomatic patients. It underscores the need for early imaging, multidisciplinary collaboration, and individualized management to prevent ascending infection and optimize outcomes.

## Introduction

The ventriculoperitoneal shunt (VPS) is the most frequently used modality for treating hydrocephalus, diverting cerebrospinal fluid (CSF) from the ventricles to the peritoneal cavity to reduce intracranial pressure [1]. These shunts typically consist of three segments: the proximal catheter originating within the ventricles, the reservoir connected to a unidirectional valve, and the distal catheter, which terminates in the peritoneal cavity [2].

The incidence of abdominal complications from VPS varies widely, ranging from 5% to 50% in the literature [1]. These most commonly include abdominal pseudocysts, ascites, and shunt migration. Spontaneous bowel perforation is a rare complication of VPS, reported in the literature as occurring in 0.01-0.07% of patients, with a reported mortality of up to 15-18% [3]. Even more rarely does bowel perforation present with transanal catheter protrusion [3]. While most cases have been reported in children, there are few case series and reports describing spontaneous bowel perforation in adults [3]. Importantly, differences in physiology, comorbidities, clinical presentation, and management considerations between adult and pediatric patients may influence both diagnosis and treatment, underscoring the need to examine these populations separately. Prompt recognition and management are essential to prevent ascending infection and other serious complications.

To better characterize this rare complication in the adult population, we present a case of spontaneous VPS-associated bowel perforation presenting with transanal catheter protrusion and perform a focused review of the literature.

## Case presentation

**Figure 1:**
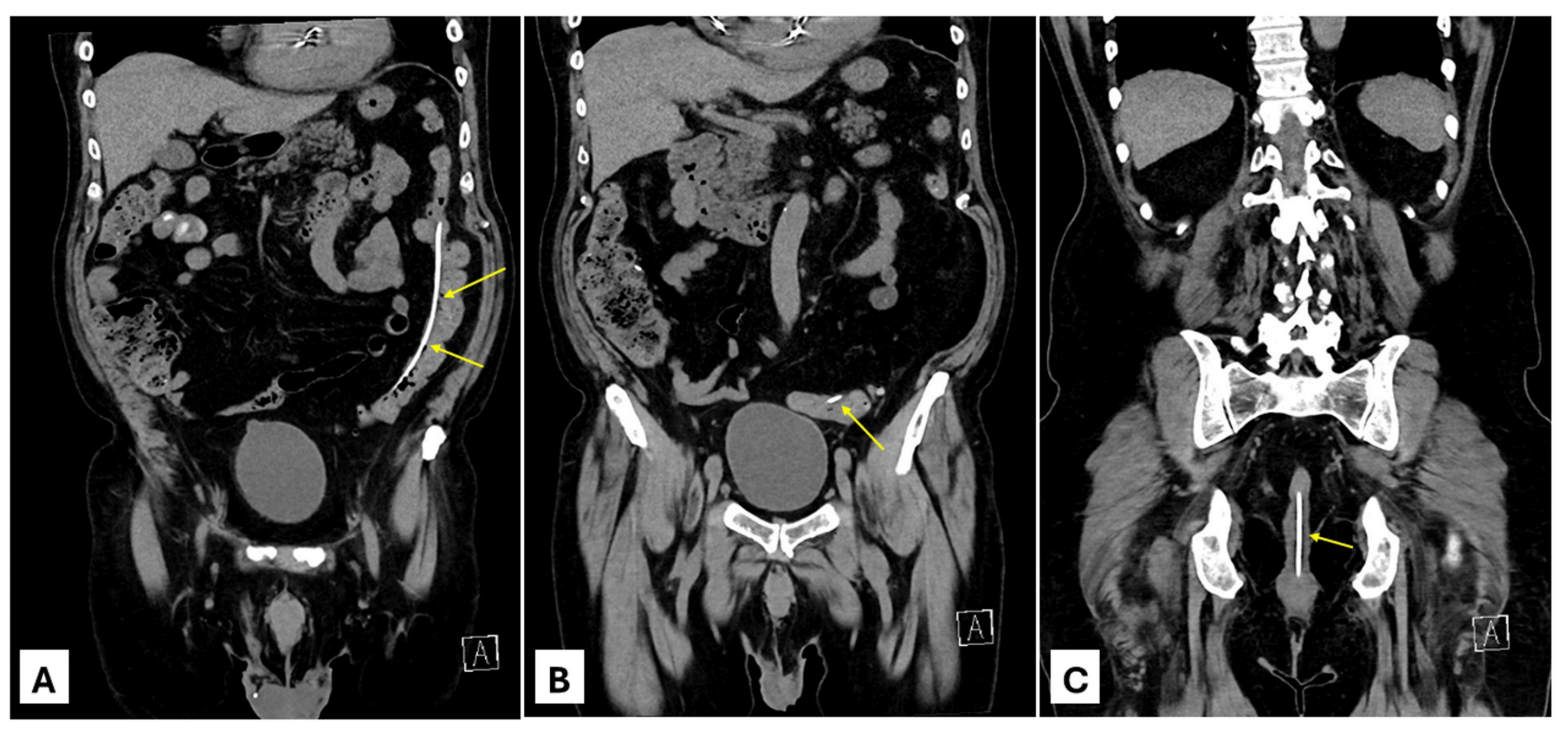
Coronal CT images at presentation to the emergency department. Yellow arrows indicate the distal catheter. (A) VPS tubing adjacent to the descending colon. (B) Shunt tubing perforating into the lumen of the descending colon. (C) Shunt tubing within the lumen of the rectum. Notably, there is no evidence of peritoneal free fluid or pneumoperitoneum. CT: computed tomography, VPS: ventriculoperitoneal shunt

An 81-year-old male with a past medical history of normal pressure hydrocephalus who underwent VPS placement six months prior, atrial fibrillation not on anticoagulation, sick sinus syndrome s/p pacemaker, benign prostatic hyperplasia, ulcerative colitis on mesalamine, and hypertension presented to the emergency department with 2 feet of narrow tubing protruding from the anus. He also noted intermittent dizziness and reported difficulty concentrating over the preceding two months. Family members confirmed noting some difficulty concentrating and intermittent somnolence when visiting him over the same time period. Review of systems was notably negative for fever, headaches, abdominal pain, nausea, and diarrhea. He was noted to be afebrile and normotensive. Work-up in the emergency department was notable for a normal white blood cell count of 7.4 with a normal absolute neutrophil count, normal serum electrolytes, and normal creatinine levels. The electrocardiogram showed a normal sinus rhythm. An X-ray VPS series was obtained first, demonstrating a well-positioned right-sided ventriculostomy shunt catheter with intact tubing in the neck, chest, abdomen, and pelvis. The CT head was negative for acute abnormality, with the right frontal approach catheter in place and mild ventriculomegaly, stable from prior CT images. The CT abdomen and pelvis was notable for a VPS catheter tubing in place extending from the lower midline chest wall into the peritoneal cavity and appearing to perforate into the lumen of the mid-descending colon before extruding out of the anus (Figure 1).

The patient was initiated on meropenem and vancomycin and was admitted to neurology with consultation to neurosurgery and general surgery, who planned for VPS removal and possible partial colectomy in the operating room the following morning. Given the patient's clinical stability, lack of infectious signs or symptoms, and the plan to treat empirically with broad-spectrum antibiotics, as discussed with the infectious diseases team, CSF sampling was not performed at that time.

The following morning, the patient first underwent upper midline laparotomy. At that time, the distal portion of the catheter was noted to be entering the mesenteric side of the left lateral portion of the descending colon. The catheter was cut at the abdominal wall, and the proximal portion of the shunt and the valve, along with the tunneled portion of the distal catheter, were removed without difficulty by the neurosurgery team. The distal portion of the descending colon was densely adherent to the catheter, requiring some sharp dissection. Once the catheter was freed, it was noted to be entering the colon at a single point in the mid-descending colon. A stay suture was placed just adjacent to the entry side, and the remainder of the distal portion of the shunt was removed through the abdomen. The colon defect was debrided back to healthy mucosa with pinpoint bleeding, and the defect was repaired primarily and oversewn. The catheter was sent for culture, which ultimately grew *Enterococcus faecalis* and *Staphylococcus haemolyticus*. The infectious diseases team recommended one week of meropenem and two weeks of vancomycin, which the patient received. The remainder of the patient’s post-operative course was unremarkable, and he was subsequently discharged in stable condition following completion of his antibiotic course.

## Discussion

A literature search was conducted to identify published cases of spontaneous bowel perforation associated with VPS placement in adult patients presenting with transanal catheter protrusion. A comprehensive search of PubMed, Embase, and Google Scholar was performed for database inception through January 2026 using combinations of the terms “ventriculoperitoneal shunt,” “bowel perforation,” “intestinal perforation," “transanal protrusion,” and “catheter migration.” Only reports describing patients aged 18 or older at the time of catheter placement who presented with transanal catheter protrusion were included. Pediatric cases, cases involving iatrogenic perforation, and review articles without individual patient data were excluded. Thirty individual cases were identified. References of included articles were manually reviewed to identify additional relevant reports. Data extracted included patient age at the time of VPS placement, presenting symptoms, management strategies, time from shunt placement to diagnosis, and clinical outcome. Spontaneous bowel perforation is a rare complication of VPS placement with a high associated mortality rate [3]. Most published reviews that examine VPS-associated bowel perforation combine pediatric and adult populations [3]. However, this approach may not be appropriate, as important differences in bowel wall thickness, immune system maturity, and prior abdominal surgery may influence the risk, clinical presentation, and outcomes of this complication. A subset of existing case reports and series describe this rare complication occurring following VPS placement in adults, in whom presentation appears to be more indolent, with some patients presenting incidentally without evidence of abdominal pathology. Here we present one such case of an 81-year-old patient presenting with transanal catheter protrusion with a benign abdominal exam who was ultimately found to have catheter-associated perforation of the descending colon. Consistent with this observation, our review of 11 cases of perforation following VPS placement in adults presenting with transanal catheter protrusion demonstrates that a substantial proportion of patients present without abdominal symptoms.

Risk factors for VPS-associated bowel perforation remain largely speculative, given the rarity of this complication. In adults, for whom cases are reported much less frequently than in children, potential risk factors include conditions that compromise the integrity of the bowel wall, such as diverticulosis, chronic constipation, chronic inflammation, or previous abdominal surgery, as well as steroid use [4,5]. Our case describes a patient with a history of ulcerative colitis, which may have placed him at higher risk for perforation given these concerns. The exact mechanism of perforation remains unclear. However, chronic irritation and pressure necrosis from the distal catheter tip may result in gradual erosion through the bowel wall, with fibrous encapsulation preventing leakage of enteric contents into the peritoneal cavity. This mechanism is supported by the relatively high proportion of asymptomatic or minimally symptomatic patients identified in our review, suggesting a gradual and contained perforation process rather than an acute event. In cases such as ours, wherein patients are without overt signs of peritonitis, this hypothesis could explain the asymptomatic presentation.

Catheter migration is a phenomenon relatively well described in the neurosurgical literature. Allouh et al. proposed a useful framework for considering patterns of migration based on catheter position: internal migration wherein the catheter invades the organs within the thoracic, abdominal, or pelvic cavities; external migration wherein the catheter penetrates through the abdominal wall into the subcutaneous tissue or exits the body entirely; and compound migration wherein the catheter perforates an existing hollow viscus organ and protrudes through an existing anatomical orifice (as in this case) [2]. Compound catheter migration has been reported in the setting of transoral, transanal, transurethral, and transvaginal protrusion. Our literature review demonstrates that transanal extrusion represents one of the most common presenting findings in adults with compound migration, often serving as the initial (or only) indication of bowel perforation. Most reported sites of perforation are the colon, with the stomach, small bowel, and rectum reported much less frequently [6]. Most reports do not specify the location of the colon; however, in our case, the descending colon was the site of perforation. The increased incidence of colonic perforation compared to other anatomic locations may be related to the fixed position of the ascending and descending colons. Alternatively, perforation of the sigmoid colon may be related to the proposed association between spontaneous perforation and diverticulosis in the adult population.

Management of these patients should be guided by clinical presentation. The primary concern for catheter perforation into a hollow viscus is for ascending infection and meningitis caused by enteric organisms [7-9]. Most, but not all, reports describe obtaining CSF cultures and a prolonged course of broad-spectrum intravenous antibiotics to manage or prevent this feared complication, even without overt neurological symptoms or signs on exam, given the high reported mortality [4,9]. Patients with peritonitis, sepsis, or neurological complications generally require emergent laparotomy with bowel repair or resection and shunt externalization. In contrast, stable patients without peritoneal signs may be managed less invasively. As demonstrated in our review, a range of management strategies have been successfully employed in adults, including shunt externalization, laparoscopic distal catheter removal, and endoscopic retrieval with generally favorable outcomes in appropriately selected patients. One case report describes stable, asymptomatic patients without signs or symptoms of infection who were managed laparoscopically with shunt externalization and distal catheter removal [9]. Several other reports also describe successful endoscopic management, in which the distal catheter is removed transanally, and clips are used to seal the perforation [4].

Although most reported cases of VPS-associated bowel perforation are described in neurosurgical literature, general surgeons can play a critical role in both the placement of ventriculoperitoneal shunts and the management of their complications [10]. In many institutions, general surgeons are involved in the abdominal portion of VPS placement and are frequently consulted when intra-abdominal complications are suspected [10]. Furthermore, patients with catheter migration or bowel perforation may initially present to general surgery services, particularly when catheter extrusion, abdominal imaging abnormalities, or concern for viscus perforation is identified. As such, general surgeons need to be aware of this rare but serious complication, recognize its often subtle or asymptomatic presentation in adults, and understand the range of management strategies, including shunt externalization, endoscopic or laparoscopic catheter removal, and exploratory laparotomy when indicated. Based on the limited available literature, we recommend considering multidisciplinary collaboration to optimize outcomes and reduce morbidity and mortality. We recognize, however, that these case reports are heterogenous in their management strategies, and any conclusions drawn are inherently limited by the observational data currently available (Table 1).

**Table 1:** Cases of spontaneous bowel perforation by VPSs placed in adults presenting with transanal catheter protrusion VPSs: ventriculoperitoneal shunts

Case number	Author(s)	Age at time of placement	Presentation	Management	Time from placement to diagnosis	Outcome
1	Yousfi et al., 2003 [5]	24	Catheter protruding per anus	Distal catheter removed endoscopically	Unknown	Recovery
2	Nishijima et al., 1982 [11]	69	Catheter protruding per anus	Systemic and intrathecal antibiotics, catheter left in place	2 years	Died from pneumonia
3	Brownlee et al., 1998 [12]	40	Catheter protruding per anus	Proximal catheter removed, distal portion cut, and left to be eliminated during defecation	3 months	Recovery
4	Pikoulis et al., 2003 [13]	33	Catheter protruding per anus	Proximal catheter removed, distal catheter removed endoscopically	6 months	Recovery
5	Martinez Hernandez-Magro et al., 2006 [14]	34	Catheter protruding per anus	Shunt externalized, distal catheter removed via laparotomy	2 years	Recovery
6	Doglietto et al., 2006 [15]	36	Headache, fever, catheter protrusion per anus	Shunt externalized, distal catheter cut and left to be eliminated during defecation	2 years	Recovery
7	Hayama et al., 2011 [16]	39	Catheter protruding per anus	Proximal catheter removed, distal catheter removed via laparotomy	2 years	Recovery
8	Perez-Aguado et al., 2021 [17]	87	Catheter protruding per anus	Shunt externalized, distal catheter removed endoscopically	unknown	Recovery
9	Mirjalali et al., 2024 [18]	36	Headaches, supraclavicular swelling, catheter protrusion per anus	Proximal catheter cut and removed at the neck, distal catheter removed from the anus	2 years	Recovery
10	Allsbrook et al., 2024 [19]	29	Headache, epigastric pain, diarrhea, catheter protrusion per anus	Shunt externalized, distal catheter removed laparoscopically	Unknown	Recovery
11	Pillai et al., 2025 [20]	21	Catheter protrusion per anus	Shunt externalized, distal catheter removed via laparotomy	6 months	Recovery

## Conclusions

VPS-associated bowel perforation is a rare but potentially life-threatening complication that may present without abdominal or neurological symptoms in adult patients. Transanal catheter protrusion may represent the initial clinical manifestation. Prompt recognition, imaging, surgical consultation, and appropriate management are recommended to prevent infectious complications. This case contributes to the limited literature describing asymptomatic bowel perforation in elderly patients. It underscores the importance of maintaining clinical vigilance in patients with VPS presenting with transanal catheter extrusion.
